# Supplementary material for: Polyploids broadly generate novel haplotypes from trans-specific variation in Arabidopsis arenosa and Arabidopsis lyrata
Source: PLoS Genet. 2024 Dec 23;20(12):e1011521. doi: 10.1371/journal.pgen.1011521 (PMC11706510; doi:10.1371/journal.pgen.1011521)
Supplement: S1 Table — (DOCX) [file pgen.1011521.s008.docx]

| ‍pop | ‍ploidy | num_snps | num_sites | num_singletons | nucleotide_diversity | Tajima’s_D |
| --- | --- | --- | --- | --- | --- | --- |
| ‍VLH | ‍2 | 110,098 | 3,443,207 | 37,098 | 0.013 | 0.10 |
| ‍OSL | 2 | 93,965 | 3,419,890 | 26,254 | 0.011 | 0.29 |
| ‍STD | 2 | 108,857 | 3,823,332 | 30,475 | 0.012 | 0.30 |
| ‍JOH | 4 | 215,901 | 3,861,780 | 49,715 | 0.018 | 0.27 |
| ‍MOD | 4 | 222,924 | 3,815,757 | 57,494 | 0.018 | 0.09 |
| ‍PEK | ‍4 | 166,466 | 3,663,712 | 34,317 | 0.015 | 0.34 |
| ‍TEM | ‍4 | 183,885 | 3,867,146 | 41,646 | 0.015 | 0.27 |
| ‍SCT | 4 | 161,092 | 3,881,388 | 34,141 | 0.014 | 0.38 |

*num_snps: number of SNPs in the analysis, num_sites: number of genomic sites considered in the analysis. Nucleotide diversity and Tajima’s D for A. arenosa populations are reported in [26].*
