# Supplementary material for: Polyploids broadly generate novel haplotypes from trans-specific variation in Arabidopsis arenosa and Arabidopsis lyrata
Source: PLoS Genet. 2024 Dec 23;20(12):e1011521. doi: 10.1371/journal.pgen.1011521 (PMC11706510; doi:10.1371/journal.pgen.1011521)
Supplement: S2 Table — (DOCX) [file pgen.1011521.s009.docx]

| Method | A. lyrata  code | A. thaliana code | Name | TAIR annotation | N lineages candidate SNPs | N lineages PicMin | PicMin FDR corrected p-value |
| --- | --- | --- | --- | --- | --- | --- | --- |
| candidate SNPs | AL1G26770 | AT1G14750.1 | SDS | Encodes a meiotic cyclin-like protein, distinct from all other known Arabidopsis cyclins. It is not required for meiotic DSB formation but is necessary for meiotic DSB repair via the homologous chromosome. | 4 |  |  |
| PicMin + candidate SNPs | AL1G27690 | AT1G15570.1 | CYCA2;3 | A2-type cyclin. Negatively regulates endocycles and acts as a key regulator of ploidy levels in Arabidopsis endoreduplication. Interacts physically with CDKA;1. Expressed preferentially in trichomes and young developing tissues. | 4 | 4 | 0.00170 |
| PicMin + candidate SNPs | AL1G35730 | AT1G22275.1 | ZYP1b | One of two nearly identical proteins (ZYP1a) identified by similarity to transverse filament (TF) proteins. These proteins are involved in chromosome synapsis during meiosis I and localize to the synaptonemal complex (SC). Single mutants have reduced fertility and double mutants (induced by RNAi) have severely reduced fertility. | 4 | 4 | 0.00170 |
| PicMin + candidate SNPs | AL1G56960 | AT1G49590.1 | ZOP1 | Encodes a novel nucleic acid-binding protein that is required for both RdDM (RNA-directed DNA methylation) and pre-mRNA splicing. | 4 | 4 | 0.01552 |
| candidate SNPs | AL1G62040 | AT1G53490.1 | HEI10 | Encodes HEI10, a RING finger-containing protein. HEI10 belongs to a group of proteins well conserved among species known as ZMM. Required for class I crossover formation. The mRNA is cell-to-cell mobile. | 4 |  |  |
| PicMin + candidate SNPs | AL2G25920 | AT1G67370.1 | ASY1 | meiotic asynaptic mutant 1 (ASY1). ASY1 protein is initially distributed as numerous foci throughout the chromatin. During early G2, the foci are juxtaposed to the nascent chromosome axes to form a continuous axis associated signal. | 4 | 4 | 0.00170 |
| PicMin + candidate SNPs | AL2G37810 | AT1G77600.2 | PDS5b | One of 5 PO76/PDS5 cohesion cofactor orthologs of Arabidopsis. | 4 | 3 | 0.05756 |
| PicMin + candidate SNPs | AL3G24370 | AT3G12690.2 | AGC1.5 | Encodes a putative serine/threonine kinase It is expressed specifically in pollen and appears to function redundantly with AGC1.7 to regulate polarized growth of pollen tubes. | 3 | 3 | 0.04452 |
| PicMin + candidate SNPs | AL3G29960 | AT3G16980.1 | NRPB9A | One of two highly similar, non-catalytic subunits common to nuclear DNA-directed RNA polymerases II, IV and V; homologous to budding yeast RPB9. Appears to be redundant with At4g16265 | 3 | 3 | 0.01509 |
| PicMin + candidate SNPs | AL4G29630 | AT2G33845.1 | AT2G33845 | mRNA-binding, OB-fold-like protein, cell to cell mobile | 4 | 4 | 0.00226 |
| PicMin + candidate SNPs | AL4G46460 | AT2G46980.2 | ASY3 | Encodes ASY3, a coiled-coil domain protein that is required for normal meiosis. | 3 | 3 | 0.00255 |
| PicMin + candidate SNPs | AL5G32860 | AT3G52270.1 | TfIIFbeta | Transcription initiation factor IIF, beta subunit; transcription initiation from RNA polymerase II promoter | 4 | 4 | 0.00170 |
| candidate SNPs | AL6G15380 | AT5G05490.2 | SYN1/REC8 | Encodes a RAD21-like gene essential for meiosis. Encodes a 627 a.a. protein that is slightly longer in the N-terminus than SYN1 BP5. | 2 |  |  |
| PicMin + candidate SNPs | AL7G13140 | AT4G37630.1 | CYCD5;1 | core cell cycle genes; a quantitative trait gene for endoreduplication. | 4 | 3 | 0.02843 |
| PicMin + candidate SNPs | AL7G35790 | AT4G18490.2 | AT4G18490 | unknown protein; expressed in flower stage 15-18, co-transcription with CYCA2;3 | 4 | 4 | 0.00170 |
| PicMin + candidate SNPs | AL8G25600 | AT5G51340.1 | SCC4 | SCC4 is a tetratricopeptide repeat containing protein and a likely component of a plant cohesion loading complex along with its partner SSC2 It is expressed primarily in dividing cells. Loss of function mutants are embryo lethal, arresting by globular stage. | 3 | 4 | 0.00611 |
| PicMin + candidate SNPs | AL8G44080 | AT5G67260.1 | CYCD3;2 | Encode CYCD3;2, a CYCD3 D-type cyclin. Important for determining cell number in developing lateral organs. Mediating cytokinin effects in apical growth and development. | 3 | 3 | 0.07481 |
