# Supplementary material for: Polyploids broadly generate novel haplotypes from trans-specific variation in Arabidopsis arenosa and Arabidopsis lyrata
Source: PLoS Genet. 2024 Dec 23;20(12):e1011521. doi: 10.1371/journal.pgen.1011521 (PMC11706510; doi:10.1371/journal.pgen.1011521)
Supplement: S3 Table — (DOCX) [file pgen.1011521.s010.docx]

| Category | Term ID | Term description | Observed gene count | Background gene count | Strength | False discovery rate | Matching proteins in your network (labels) |
| --- | --- | --- | --- | --- | --- | --- | --- |
| GO Process | GO:0007049 | Cell cycle | 10 | 489 | 1.52 | 2.96E-10 | SDS,CYCA2;3,ZYP1b,HEI10,ASY1,ASY3,CYCD5;1,SYN1,AT5G51340,CYCD3;2 |
| GO Process | GO:0022402 | Cell cycle process | 9 | 333 | 1.64 | 3.43E-10 | SDS,CYCA2;3,ZYP1b,HEI10,ASY1,CYCD5;1,SYN1,AT5G51340,CYCD3;2 |
| GO Process | GO:0098813 | Nuclear chromosome segregation | 6 | 72 | 2.13 | 8.22E-09 | SDS,ZYP1b,HEI10,ASY1,SYN1,AT5G51340 |
| GO Process | GO:0045143 | Homologous chromosome segregation | 5 | 28 | 2.46 | 1.32E-08 | SDS,ZYP1b,HEI10,ASY1,SYN1 |
| GO Process | GO:0000280 | Nuclear division | 6 | 113 | 1.93 | 5.47E-08 | SDS,ZYP1b,HEI10,ASY1,SYN1,AT5G51340 |
| GO Process | GO:0070192 | Chromosome organization involved in meiotic cell cycle | 5 | 43 | 2.27 | 5.47E-08 | SDS,ZYP1b,HEI10,ASY1,SYN1 |
| GO Process | GO:0051321 | Meiotic cell cycle | 6 | 157 | 1.79 | 2.01E-07 | SDS,ZYP1b,HEI10,ASY1,ASY3,SYN1 |
| GO Process | GO:0007129 | Homologous chromosome pairing at meiosis | 4 | 23 | 2.45 | 6.35E-07 | SDS,ZYP1b,HEI10,ASY1 |
| GO Process | GO:0007131 | Reciprocal meiotic recombination | 4 | 45 | 2.16 | 6.18E-06 | SDS,ZYP1b,HEI10,ASY1 |
| GO Process | GO:0044772 | Mitotic cell cycle phase transition | 4 | 45 | 2.16 | 6.18E-06 | SDS,CYCA2;3,CYCD5;1,CYCD3;2 |
| GO Process | GO:1903047 | Mitotic cell cycle process | 5 | 148 | 1.74 | 6.97E-06 | SDS,CYCA2;3,CYCD5;1,AT5G51340,CYCD3;2 |
| GO Process | GO:0022414 | Reproductive process | 9 | 1429 | 1.01 | 1.08E-05 | SDS,ZYP1b,HEI10,ASY1,ASY3,AGC1.5,SYN1,AT5G51340,CYCD3;2 |
| GO Process | GO:0051301 | Cell division | 6 | 348 | 1.44 | 1.08E-05 | SDS,CYCA2;3,ZYP1b,CYCD5;1,AT5G51340,CYCD3;2 |
| GO Process | GO:0006259 | DNA metabolic process | 6 | 378 | 1.41 | 1.50E-05 | SDS,ZYP1b,HEI10,ASY1,NRPB9A,SYN1 |
| GO Process | GO:0051026 | Chiasma assembly | 3 | 12 | 2.61 | 1.54E-05 | SDS,HEI10,ASY1 |
| GO Process | GO:0051276 | Chromosome organization | 6 | 487 | 1.3 | 5.43E-05 | SDS,ZYP1b,HEI10,ASY1,SYN1,AT5G51340 |
| GO Process | GO:0051726 | Regulation of cell cycle | 5 | 244 | 1.52 | 5.43E-05 | SDS,CYCA2;3,ASY3,CYCD5;1,CYCD3;2 |
| GO Process | GO:0090304 | Nucleic acid metabolic process | 7 | 1403 | 0.91 | 0.0014 | SDS,ZYP1b,AT1G49590,HEI10,ASY1,NRPB9A,SYN1 |
| GO Process | GO:0010444 | Guard mother cell differentiation | 2 | 11 | 2.47 | 0.0031 | CYCA2;3,CYCD3;2 |
| GO Process | GO:0042023 | DNA endoreduplication | 2 | 19 | 2.23 | 0.0081 | CYCA2;3,CYCD5;1 |
| GO Process | GO:0007062 | Sister chromatid cohesion | 2 | 24 | 2.13 | 0.0119 | SYN1,AT5G51340 |
| GO Process | GO:0044260 | Cellular macromolecule metabolic process | 9 | 3665 | 0.6 | 0.012 | SDS,CYCA2;3,ZYP1b,HEI10,ASY1,AGC1.5,NRPB9A,CYCD5;1,SYN1 |
| GO Process | GO:0043170 | Macromolecule metabolic process | 10 | 4720 | 0.53 | 0.0132 | SDS,CYCA2;3,ZYP1b,AT1G49590,HEI10,ASY1,AGC1.5,NRPB9A,CYCD5;1,SYN1 |
| GO Process | GO:0006281 | DNA repair | 3 | 260 | 1.27 | 0.042 | SDS,NRPB9A,SYN1 |
| GO Function | GO:0016538 | Cyclin-dependent protein serine/threonine kinase regulator activity | 4 | 52 | 2.09 | 7.64E-05 | SDS,CYCA2;3,CYCD5;1,CYCD3;2 |
| GO Component | GO:0000794 | Condensed nuclear chromosome | 4 | 27 | 2.38 | 2.57E-06 | ZYP1b,HEI10,ASY1,SYN1 |
| GO Component | GO:0000307 | Cyclin-dependent protein kinase holoenzyme complex | 4 | 48 | 2.13 | 8.39E-06 | SDS,CYCA2;3,CYCD5;1,CYCD3;2 |
| GO Component | GO:0005694 | Chromosome | 6 | 300 | 1.51 | 8.39E-06 | ZYP1b,HEI10,ASY1,ASY3,SYN1,AT5G51340 |
| GO Component | GO:0061695 | Transferase complex, transferring phosphorus-containing groups | 5 | 169 | 1.68 | 9.24E-06 | SDS,CYCA2;3,NRPB9A,CYCD5;1,CYCD3;2 |
| GO Component | GO:0005634 | Nucleus | 13 | 4669 | 0.65 | 1.26E-05 | SDS,CYCA2;3,ZYP1b,AT1G49590,HEI10,ASY1,ASY3,AGC1.5,NRPB9A,CYCD5;1,SYN1,AT5G51340,CYCD3;2 |
| GO Component | GO:0031981 | Nuclear lumen | 7 | 807 | 1.15 | 2.70E-05 | ZYP1b,AT1G49590,HEI10,ASY1,ASY3,NRPB9A,SYN1 |
| GO Component | GO:0043232 | Intracellular non-membrane-bounded organelle | 7 | 1407 | 0.9 | 0.00064 | ZYP1b,HEI10,ASY1,ASY3,NRPB9A,SYN1,AT5G51340 |
| GO Component | GO:0032991 | Protein-containing complex | 8 | 2226 | 0.76 | 0.0011 | SDS,CYCA2;3,AT1G49590,NRPB9A,CYCD5;1,SYN1,AT5G51340,CYCD3;2 |
| GO Component | GO:0042025 | Host cell nucleus | 3 | 150 | 1.51 | 0.0047 | SDS,CYCA2;3,CYCD3;2 |
| GO Component | GO:0005654 | Nucleoplasm | 3 | 396 | 1.09 | 0.0472 | AT1G49590,ASY1,NRPB9A |
| UniProt Keywords | KW-0469 | Meiosis | 6 | 70 | 2.14 | 2.09E-09 | SDS,ZYP1b,HEI10,ASY1,ASY3,SYN1 |
| UniProt Keywords | KW-0131 | Cell cycle | 7 | 266 | 1.63 | 3.74E-08 | SDS,CYCA2;3,ZYP1b,CYCD5;1,SYN1,AT5G51340,CYCD3;2 |
| UniProt Keywords | KW-0132 | Cell division | 6 | 198 | 1.69 | 2.83E-07 | SDS,CYCA2;3,ZYP1b,CYCD5;1,AT5G51340,CYCD3;2 |
| UniProt Keywords | KW-0195 | Cyclin | 4 | 52 | 2.09 | 4.24E-06 | SDS,CYCA2;3,CYCD5;1,CYCD3;2 |
| UniProt Keywords | KW-0158 | Chromosome | 3 | 117 | 1.62 | 0.005 | HEI10,ASY1,ASY3 |
| UniProt Keywords | KW-0159 | Chromosome partition | 2 | 24 | 2.13 | 0.0091 | SYN1,AT5G51340 |
| UniProt Keywords | KW-0539 | Nucleus | 9 | 3770 | 0.59 | 0.0101 | CYCA2;3,ZYP1b,AT1G49590,HEI10,ASY1,ASY3,NRPB9A,SYN1,AT5G51340 |
| STRING clusters | CL:6994 | Cell division, and microtubule-based movement | 7 | 164 | 1.84 | 3.22E-08 | SDS,CYCA2;3,AT1G77600,CYCD5;1,SYN1,AT5G51340,CYCD3;2 |
| STRING clusters | CL:7443 | Homologous chromosome pairing at meiosis, and meiosis protein spo22/zip4 like | 4 | 13 | 2.7 | 6.45E-07 | ZYP1b,HEI10,ASY1,ASY3 |
| STRING clusters | CL:7001 | Cyclin, and RNA polymerase II CTD heptapeptide repeat kinase activity | 4 | 38 | 2.23 | 1.20E-05 | SDS,CYCA2;3,CYCD5;1,CYCD3;2 |
| STRING clusters | CL:7270 | Cohesin complex, and smc loading complex | 3 | 10 | 2.68 | 4.34E-05 | AT1G77600,SYN1,AT5G51340 |
| STRING clusters | CL:7005 | Cyclins are a family of proteins that control the progression of cells through the cell cycle by activating cyclin-dependent kinase (Cdk) enzymes., and Cyclin-dependent kinase regulatory subunit | 3 | 23 | 2.32 | 0.00027 | CYCA2;3,CYCD5;1,CYCD3;2 |
| STRING clusters | CL:7007 | Cyclins are a family of proteins that control the progression of cells through the cell cycle by activating cyclin-dependent kinase (Cdk) enzymes., and regulation of stomatal complex patterning | 2 | 12 | 2.43 | 0.0126 | CYCA2;3,CYCD3;2 |
