# Supplementary material for: Polyploids broadly generate novel haplotypes from trans-specific variation in Arabidopsis arenosa and Arabidopsis lyrata
Source: PLoS Genet. 2024 Dec 23;20(12):e1011521. doi: 10.1371/journal.pgen.1011521 (PMC11706510; doi:10.1371/journal.pgen.1011521)
Supplement: S4 Table — AF: allele frequency of haplotype. (DOCX) [file pgen.1011521.s011.docx]

| Gene | AF (tetraploid) | AF  (A. arenosa diploid) | AF  (A. lyrata diploid) | Other | Tetraploid haplotype in N populations | Total N of tetraploid populations | Presence |
| --- | --- | --- | --- | --- | --- | --- | --- |
| CYCA2_3 | 0.47 | 0.02 | 0.04 | 0.48 | 56 | 61 | 91.8% |
| CYCD3_2 | 0.61 | 0.13 | 0.06 | 0.20 | 59 | 60 | 98.3% |
| CYCD5_1 | 0.52 | 0.12 | 0.07 | 0.30 | 56 | 60 | 93.3% |
| AT4G18490 | 0.57 | 0.10 | 0.04 | 0.30 | 57 | 60 | 95.0% |
| ZYP1b | 0.68 | 0.05 | 0.08 | 0.18 | 57 | 58 | 98.3% |
| ASY1 | 0.62 | 0.06 | 0.02 | 0.31 | 57 | 60 | 95.0% |
| PDS5b | 0.73 | 0.02 | 0.02 | 0.23 | 58 | 60 | 96.7% |
| ASY3 | 0.61 | 0.01 | 0.01 | 0.36 | 56 | 59 | 94.9% |
| SCC4 | 0.57 | 0.16 | 0.03 | 0.23 | 59 | 61 | 96.7% |
| NRPB9A | 0.73 | 0.06 | 0.02 | 0.19 | 47 | 50 | 94.0% |
| TfIIFbeta | 0.85 | 0.03 | 0.01 | 0.12 | 55 | 58 | 94.8% |
| AT2G33845 | 0.51 | 0.04 | 0.02 | 0.42 | 58 | 60 | 96.7% |
