# Supplementary material for: Polyploids broadly generate novel haplotypes from trans-specific variation in Arabidopsis arenosa and Arabidopsis lyrata
Source: PLoS Genet. 2024 Dec 23;20(12):e1011521. doi: 10.1371/journal.pgen.1011521 (PMC11706510; doi:10.1371/journal.pgen.1011521)
Supplement: S5 Table — The proportion of SNPs representing each of the seven source scenarios is given (see Fig 4A for graphical visualization of scenarios). (DOCX) [file pgen.1011521.s012.docx]

| 1000 bootstraps of 232 synonymous SNPs | | | | | | | 232 candidate SNPs |
| --- | --- | --- | --- | --- | --- | --- | --- |
| Scenario | Mean | Sem | Lower | Upper | Min | Max | Total |
| 1 | 43.6% | 0.14% | 43.4% | 43.7% | 27.2% | 59.1% | 28.9% |
| 2 | 5.5% | 0.07% | 5.4% | 5.6% | 0.4% | 17.2% | 29.3% |
| 3 | 0.3% | 0.02% | 0.3% | 0.3% | 0% | 3.5% | 3.0% |
| 4 | 0.6% | 0.02% | 0.6% | 0.7% | 0% | 4.7% | 4.3% |
| 5 | 23.7% | 0.13% | 23.6% | 23.8% | 11.6% | 37.5% | 6.5% |
| 6 | 1.8% | 0.04% | 1.8% | 1.9% | 0% | 6.9% | 0.4% |
| 7 | 24.5% | 0.12% | 24.3% | 24.6% | 12.1% | 36.2% | 27.6% |
| Trans-specific | 50.0% |  | | | | | |
| Introgression | 56.4% |  |  |  |  |  |  |
| De novo | 24.5% |  |  |  |  |  |  |
