# Supplementary material for: Polyploids broadly generate novel haplotypes from trans-specific variation in Arabidopsis arenosa and Arabidopsis lyrata
Source: PLoS Genet. 2024 Dec 23;20(12):e1011521. doi: 10.1371/journal.pgen.1011521 (PMC11706510; doi:10.1371/journal.pgen.1011521)
Supplement: S1 Text — (DOCX) [file pgen.1011521.s022.docx]

**Supplementary Text 1**

The 17 tetraploid positively selected genes (PSGs) identified in this study are involved in a range of processes, including homologous chromosome pairing during prophase I of meiosis, cell cycle timing and regulation of endoreduplication through the action of different types of cyclins, and mRNA transcription via RNA polymerase II. ­In previous research, the role of meiosis genes in tetraploid adaptation has been identified and tested, with results suggesting that these functional changes contribute to the successful establishment of tetraploids [1–4]⁠. Cell cycle regulation has also been implied in relation to polyploid adaptation [1, 5]. Cyclins D1 and D2 have been shown to promote tolerance to genome doubling in tumors [6, 7]⁠, while cyclin A2;3 regulates the extent of endoreduplication in *A. thaliana* [8]⁠, and cyclin D5;1 determines the rate of endoreduplication [9]⁠. These findings suggest that cyclins may play a role in the adaptation to whole genome duplication (WGD), potentially by regulating endoreduplication levels, which further on influences the trade-off of cell size and cell number [10]. A role of transcription-regulating genes in the adaptation to WGD has also been proposed [11, 12]⁠, and the transcription cycle has been shown to be linked with the cell cycle, through the interaction of RNA polymerase II with cyclin-dependent kinases [13, 14]. It is possible that these genes interact to jointly re-time the cell cycle in response to WGD.

Interestingly, positive selection acts at different types of SNPs among these three processes. The selection of upstream regulatory variability was more prevalent among cyclins (45% of upstream regulatory SNPs in cyclins compared to 32% genome-wide, p = 0.16, Chi square test; S3 Fig). In contrast, selection acting on nonsynonymous SNPs in coding regions was more common in meiosis genes (40% of nonsynonymous SNPs in meiosis genes compared to 28% genome-wide, p = 0.001, Chi square test; S3 Fig). Finally, transcription genes showed positive selection on synonymous SNPs in coding regions (65% of synonymous SNPs in transcription genes compared to 27% genome-wide, p < 0.001, Chi square test; S3 Fig). These results suggest that the evolution of cyclins may have a more regulatory, possibly dosage-dependent basis, while meiosis evolution may be driven more by structural protein changes. It remains to be determined if the repeated differentiation in synonymous positions of some of the transcription genes results in any functional changes.

**References**

**1. Yant L, Hollister JD, Wright KM, Arnold BJ, Higgins JD, Franklin FCH, et al. Meiotic adaptation to genome duplication in Arabidopsis arenosa. Curr Biol. 2013;**23(21): 2151–6.

2. Morgan C, Zhang H, Henry CE, Franklin FCH, Bomblies K. Derived alleles of two axis proteins affect meiotic traits in autotetraploid Arabidopsis arenosa. Proc Natl Acad Sci U S A. 2020;117(16): 8980–88.

3. Seear PJ, France MG, Gregory CL, Heavens D, Schmickl R, Yant L, et al. A novel allele of ASY3 is associated with greater meiotic stability in autotetraploid Arabidopsis lyrata. PLoS Genet. 2020;16(7): e1008900.

4. Morgan C, Knight E, Bomblies K. The meiotic cohesin subunit REC8 contributes to multigenic adaptive evolution of autopolyploid meiosis in Arabidopsis arenosa. PLoS Genet. 2022;18(7): e1010304.

5. Bray **SM, Hämälä T, Zhou M, Busoms S, Fischer S, Desjardins SD, et al. Kinetochore and ionomic adaptation to whole-genome duplication in Cochlearia shows evolutionary convergence in three autopolyploids. Cell Rep. 2024;43(8): 114576.**

6. Potapova TA, Seidel CW, Box AC, Rancati G, Li R. Transcriptome analysis of tetraploid cells identifies cyclin D2 as a facilitator of adaptation to genome doubling in the presence of p53. Mol Biol Cell. 2016;27(20): 3065–84.

7. Crockford A, Zalmas LP, Grönroos E, Dewhurst SM, McGranahan N, Cuomo ME, et al. Cyclin D mediates tolerance of genome-doubling in cancers with functional p53. Ann Oncol. 2017;28(1): 149–56.

8. Imai KK, Ohashi Y, Tsuge T, Yoshizumi T, Matsui M, Oka A, et al. The A-type cyclin CYCA2;3 is a key regulator of ploidy levels in Arabidopsis endoreduplication. Plant Cell. 2006;18(2): 382**–**96.

9. Sterken R, Kiekens R, Boruc J, Zhang F, Vercauteren A, Vercauteren I, et al. Combined linkage and association mapping reveals CYCD5;1 as a quantitative trait gene for endoreduplication in Arabidopsis. Proc Natl Acad Sci U S A. 2012;109(12): 4678**–**83.

10. Robinson DO, Coate JE, Singh A, Hong L, Bush M, Doyle JJ, et al. Ploidy and size at multiple scales in the Arabidopsis sepal. Plant Cell. 2018;30(10): 2308–29.

11. Hollister JD, Arnold BJ, Svedin E, Xue KS, Dilkes BP, Bomblies K. Genetic adaptation associated with genome-doubling in autotetraploid Arabidopsis arenosa. PLoS Genet. 2012;8(12): e1003093.

12. Marburger S, Monnahan P, Seear PJ, Martin SH, Koch J, Paajanen P, et al. Interspecific introgression mediates adaptation to whole genome duplication. Nat Commun. 2019;10(1): 5218.

13. Bregman DB, Pestell RG, Kidd VJ. Cell cycle regulation and RNA polymerase II. Front Biosci. 2000;5: D244–57.

14. Guo Z, Stiller JW. Comparative genomics of cyclin-dependent kinases suggest co-evolution of the RNAP II C-terminal domain and CTD-directed CDKs. BMC Genom. 2004;5: 69.
