# Supplementary material for: Polyploids broadly generate novel haplotypes from trans-specific variation in Arabidopsis arenosa and Arabidopsis lyrata
Source: PLoS Genet. 2024 Dec 23;20(12):e1011521. doi: 10.1371/journal.pgen.1011521 (PMC11706510; doi:10.1371/journal.pgen.1011521)
Supplement: S2 Text — (DOCX) [file pgen.1011521.s023.docx]

**Supplementary Text 2**

Some phenotypic shifts in polyploids have been associated with the processes identified in our protein-protein interaction analysis (Fig 2F). Here, we asked if established tetraploids reduce their level of endoreduplication⁠. To do so, we grew plants from each of the four established tetraploid lineages, their closely related diploids, and synthetic (oryzalin-induced) neo-tetraploids. We found that the level of endoreduplication (i.e. the fraction of endoreduplicated nuclei) and the number of endoreduplication cycles were consistently higher in diploids compared to tetraploids (S4 Fig). Notably, established tetraploids showed reduced endoreduplication compared to neo-tetraploids (mean level of endoreduplication = 0.82/0.74/0.61 for diploids/neo-tetraploids/tetraploids, p < 0.001; median maximum number of endoreduplication cycles = 3/2.5/2 for diploids/neo-tetraploids/tetraploids, p < 0.001; Wilcoxon rank sum test). In diploids, we observed a nonsignificant difference in the number of endoreduplication cycles (median = 3 in all four diploid lineages, p > 0.05, Wilcoxon rank sum test) and only a subtle difference in the level of endoreduplication in one lineage (S5 Fig). This suggests that the regulation of endoreduplication in *Arabidopsis* is generally conserved, as long as the intrinsic cell conditions do not change, for example as a result of WGD.

In summary, these results point to compensation for DNA content per nucleus: while nearly double in neo-tetraploids, it returns toward diploid levels in established tetraploids (S4 Fig), in line with genome downsizing after a WGD event [1]. This suggests ‘compensation’ over ‘trait change’ scenarios, as has been suggested for other polyploidy-specific traits following WGD [2]⁠. The reason for such evolutionary change is unclear, but may reflect a high cost of additional rounds of genome duplication in polyploids and/or structural constraints on maximum cell size [3, 4].

*Methods:*

To investigate whether genetic changes in cyclin genes may lead to a reduction in the level of endoreduplication, we grew 10 plants from each of the four lineages, from a diploid and tetraploid population each per lineage (totaling 80 plants), and we cultivated nine oryzalin-induced neo-tetraploid plants of *A. arenosa*, generated from the Western Carpathian diploid lineage, which was shown to be the progenitor of tetraploids [5]⁠. We determined the endoreduplication profile of these plants performing flow cytometric analysis on nuclei isolated from fully grown leaves (11th leaf in the rosette; [6]⁠).

Specifically, we collected seeds from both diploid and tetraploid populations of *A. lyrata* and *A. arenosa* corresponding to their four tetraploid lineages, and stored them at 4°C for vernalization. We germinated the seeds on moist filter paper in petri dishes. For induction of synthetic polyploids, we applied a drop of oryzalin solution (5% aqueous DMSO) to the shoot apical meristems of young seedlings at the stage of fully developed cotyledons. After 48 hours, we rinsed the seedlings with distilled water and transplanted them to soil, cultivating them under controled conditions (20°C/16h day, 15°C/8h night).

To estimate the success rate of the oryzalin treatment, the ploidy level of each plant was tested by flow cytometry following [7]⁠. Specifically, 0.5 cm^2^ of leaf tissue (without the middle vein) was chopped, using a razor blade, in Otto I buffer simultaneously with the same amount of *Carex acutiformis* as internal standard. Samples were then dyed with a staining solution consisting of Otto II buffer, DAPI (4',6-diamidino-2-phenylindole), and β-mercaptoethanol, and analyzed on a CytoFLEX S flow cytometer (Beckman Coulter, Inc) equipped with a UV laser (375 nm, 60 mW) using the Beckman Coulter CytExpert Acquisition and Analysis Software v.2.5. Only the plants with no or significantly low percentage (< 5% of all events) of diploid nuclei (also showing no distinguishable population of nuclei) were considered good candidates for synthetic polyploids, and nine of them were chosen for further analysis.

To assess the level of endopolyploidy, the amount of DNA was estimated using a Partec CyFlow SL flow cytometer equipped with a 532 nm solid-state laser (Cobolt Samba 150 mW). Samples were processed using the same simplified two-step method with Otto buffers and stained with propidium iodide. Samples were analyzed without internal standard. Resulting histograms/dot plots were evaluated using Partec FloMax v.2.4d.

We calculated the level of endoreduplication as the number of endoreduplicated nuclei compared to the number of all nuclei in the analysis. We also quantified the maximum number of endoreduplication cycles that nuclei of each leaf underwent, considering only peaks supported by at least five nuclei of a given genome size. Finally, we calculated the endoreduplication intensity, measured as the weighted number of endoreduplication cycles per nucleus [EI = (0 × %2C) + (1 × %4C) + (2 × %8C) + (3 × %16C) + (4 × %32C)] [8]⁠. We tested for the difference between diploid, neo-tetraploid, and tetraploid endoreduplication levels and numbers using the Wilcoxon test in the R package ‘stats’.

**References**

**1. Leitch IJ, Bennett MD. Genome downsizing in polyploid plants. Biol J Linn Soc. 2004;82(4): 651–63.**

2. Bomblies K. When everything changes at once: finding a new normal after genome duplication. Proc R Soc Lond B Biol Sci. 2020;287(1939): 20202154.

3. De Rocher EJ, Harkins KR, Galbraith DW, Bohnert HJ. Developmentally regulated systemic endopolyploid in succulents with small genomes. Science. 1990;250(4977): 99–101.

4. Barow M. Endopolyploidy in seed plants. BioEssays. 2006;28(3): 271–81.

5. Arnold B, Kim S-T, Bomblies K. Single geographic origin of a widespread autotetraploid Arabidopsis arenosa lineage followed by interploidy admixture. Mol Biol Evol. 2015;32(6): 1382–95.

6. Wos G, Macková L, Kubíková K, **Kolář F**. Ploidy and local environment drive intraspecific variation in endoreduplication in Arabidopsis arenosa. Am J Bot. 2022;109(2): 259–71.

7. Doležel J, Greilhuber J, Suda J. Estimation of nuclear DNA content in plants using flow cytometry. Nat Protoc. 2007;2(9): 2233–44.

8. Sterken R, Kiekens R, Boruc J, Zhang F, Vercauteren A, Vercauteren I, et al. Combined linkage and association mapping reveals CYCD5;1 as a quantitative trait gene for endoreduplication in Arabidopsis. Proc Natl Acad Sci U S A. 2012;109(12): 4678**–**83.
