# Supplementary material for: Polyploids broadly generate novel haplotypes from trans-specific variation in Arabidopsis arenosa and Arabidopsis lyrata
Source: PLoS Genet. 2024 Dec 23;20(12):e1011521. doi: 10.1371/journal.pgen.1011521 (PMC11706510; doi:10.1371/journal.pgen.1011521)
Supplement: S3 Text — (DOCX) [file pgen.1011521.s024.docx]

**Supplementary Text 3**

The finding of mosaic sources of tetraploid haplotypes raises questions on the spatio-temporal context of the origin of these haplotype blocks, in particular about historical area contacts of both species, hybridization, and the timing of tetraploid establishment. Based on the available knowledge on the evolutionary history of both species and our results, we propose the following hypothesis:

After their origin and primary establishment, tetraploid *A. lyrata* lineages may have exhibited rather minor signs of adaptation in the candidate genes that are in the focus of our study, sweeping its adaptive diploid variability (3.4% of the total variation; Fig 4A, scenario 3 and 6) plus an unknown portion of de novo mutations (Fig 4A, scenario 7). In contrast to *A. lyrata*, tetraploid *A. arenosa* was found to bear a significant amount of likely adaptive variability inherited from its diploid progenitor (35.8% of the total variability is of *A. arenosa* diploid origin; Fig 4A, scenario 2 and 5). In addition, tetraploid *A. arenosa* also adapted from an unknown portion of de novo mutations (Fig 4A, scenario 7), altogether constituting a majority of the current adaptive haplotype blocks. *Arabidopsis arenosa* likely polyploidized in the Western Carpathians [1, 2], which is around 350 km apart from the current distribution range of *A. lyrata* (the spatially closest are the Czech and Austrian populations). This, and the fact that the primarily (sub)arctic species *A. lyrata* may have been more widespread under colder climate, could have led to peripatric hybridization of both species during the large Pleistocene vegetation turmoil at the glacial/interglacial boundaries in the area [3]⁠. Tetraploid-adaptive variants of tetraploid *A. lyrata* and *A. arenosa* may then have been introgressed in both directions (although introgression from *A. arenosa* to *A. lyrata* was found to be much more frequent) and recombined into the final tetraploid haplotype blocks, resulting in the currently observed mosaic haplotypes comprising SNPs of both *A. lyrata* and *A. arenosa* ancestry. These final tetraploid haplotypes likely offered a higher fitness advantage than the species-specific haplotypes, because they became pervasive in all tetraploid lineages of both species (Fig 3B) and spread across the entire natural distribution range of autotetraploid *Arabidopsis* in Europe (Fig 3A). To formally test these scenarios, we propose detailed population genomic sampling across all four tetraploid lineages and their contact zones, coupled with hierarchical demographic inference, to identify the most likely evolutionary history of this intriguing reticulate adaptation.

Further, it is a pending question why there is almost an order higher contribution of candidate SNPs of *A. arenosa* than *A. lyrata* ancestry (Fig 4A, scenario 2 and 5 vs. 3 and 6). It may partly be because diploid *A. arenosa* is more genetically variable than *A. lyrata* (pi = 0.026/0.012 for *A. arenosa*/*A. lyrata*; S2 Table, [4]⁠). Alternatively, *A. lyrata* may exhibit more tradeoffs between a diploid and tetraploid fitness optimum, although we miss any evidence supporting this idea. Finally, there might have been a higher initial proportion of SNPs of *A. lyrata* ancestry, but adaptive variation inherited from diploid *A. arenosa* or originated de novo might have presented a higher selective advantage. Also, some SNPs of *A. lyrata* origin might have become extinct in diploid *A. lyrata* after WGD, and thus being categorized as de novo or unsampled origin (this category of candidate SNPs is classified into scenario 4 and 7, Fig 4A). To answer if standing variation inherited from diploid *A. lyrata* brought a substantial improvement in the overall adaptation of tetraploids, it would help to understand the functional impact of *A. lyrata*-sourced variation. Although this goes beyond the scope of this study, we found that three of the seven likely *A. lyrata*-sourced mutations are nonsynonymous (Asp168Gly in *CYCA2;3*, Ser538Pro and Met482Thr in *ASY3*) while four are synonymous (in *PDS5b*, *SCC4*, and *ASY3*), suggesting a functional impact of some, but not all of the variants.

**References**

1. Arnold B, Kim S-T, Bomblies K. Single geographic origin of a widespread autotetraploid Arabidopsis arenosa lineage followed by interploidy admixture. Mol Biol Evol. 2015;32(6): 1382–95.

2. Monnahan P, Kolář F, Baduel P, Sailer C, Koch J, Horvath R, et al. Pervasive population genomic consequences of genome duplication in Arabidopsis arenosa. Nat Ecol Evol. 2019;3(3): 457–68.

3. Abraham V, Kuneš P, Petr L, Svobodov**á** HS, Koz**á**kov**á** R, Jamrichov**á E,** et al. A pollen-based quantitative reconstruction of the Holocene vegetation updates a perspective on the natural vegetation in the Czech Republic and Slovakia. Preslia. 2016;88(4): 409–34.

4. Marburger S, Monnahan P, Seear PJ, Martin SH, Koch J, Paajanen P, et al. Interspecific introgression mediates adaptation to whole genome duplication. Nat Commun. 2019;10(1): 5218.
