# Supplementary material for: Polyploids broadly generate novel haplotypes from trans-specific variation in Arabidopsis arenosa and Arabidopsis lyrata
Source: PLoS Genet. 2024 Dec 23;20(12):e1011521. doi: 10.1371/journal.pgen.1011521 (PMC11706510; doi:10.1371/journal.pgen.1011521)
Supplement: Attachment — Submitted filename: BohutinskaEtAl_PlosGenetics_response_041124.pdf [file pgen.1011521.s025.pdf]

Dear Dr Schmickl,

Thank you very much for submitting your Research Article entitled 'Polyploids broadly generate novel haplotypes from trans-specific variation in Arabidopsis arenosa and Arabidopsis lyrata' to PLOS Genetics.

The manuscript was fully evaluated at the editorial level and by independent peer reviewers. The reviewers appreciated the attention to an important topic but identified some concerns that we ask you address in a revised manuscript.

We therefore ask you to modify the manuscript according to the review recommendations. Your revisions should address the specific points made by each reviewer.

In addition we ask that you:
1) Provide a detailed list of your responses to the review comments and a description of the changes you have made in the manuscript.

2) Upload a Striking Image with a corresponding caption to accompany your manuscript if one is available (either a new image or an existing one from within your manuscript). If this image is judged to be suitable, it may be featured on our website. Images should ideally be high resolution, eye-catching, single panel square images. For examples, please browse our archive. If your image is from someone other than yourself, please ensure that the artist has read and agreed to the terms and conditions of the Creative Commons Attribution License. Note: we cannot publish copyrighted images.

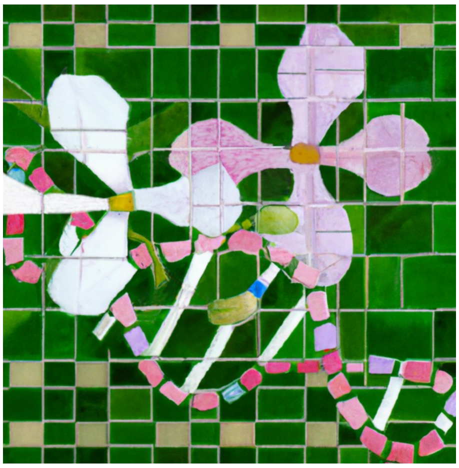

We hope to receive your revised manuscript within the next 30 days.

Please be aware that our data availability policy requires that all numerical data underlying graphs or summary statistics are included with the submission, and you will need to provide this upon resubmission if not already present. In addition, we do not permit the inclusion of phrases such as "data not shown" or "unpublished results" in manuscripts. All points should be backed up by data provided with the submission.

We added the genome-wide PicMin results underlying the PicMin Manhattan plot to Supplementary Dataset S3.

Editor's comments

The in-depth review of your manuscript by the three reviewers is now complete. Based on their assessment, it is clear that your manuscript requires some revision before it can be considered further for publication in PLOS Genetics. Comments from the external reviewers are included below.

In particular, especially please pay attention to the following issues:

Thank you for your comments. We carefully addressed all raised issues in the paragraphs in italics in this rebuttal letter and the respective parts in the main text and supplement.

1) As reviewer1 pointed out "The aspect that may seem a bit surprising to some readers and that the authors do not stress is the fact that most genome scans studies comparing diploids and tetraploids end up finding roughly the same set of genes. Those are few and related to the same general function." It is important to discuss this "Are those the only changes or are we missing the rest?".

We clarified this in our reply to Reviewer 1. We now make it very clear that the vast majority of gene candidates from each of the genome scans comparing diploids and tetraploids are not the same. Instead, studies regularly find process-level convergence (Bohutínská et al., 2021, Molecular Biology and Evolution; Bray et al., 2024, Cell Reports).

2) As reviewer2 pointed out that the importance of endopolyploidy in this study should be rephrased somehow.

We agree that endopolyploidy was not well integrated into the text. We rephrased our reasoning for detailing on endopolyploidy in both Results and Discussion (lines 171-180 and 276-281, respectively), and we added an interpretation of our results.

Reviewer's Responses to Questions

Reviewer #1:

This is an interesting and carefully carried out study.

Thank you.

The paper is well written but, perhaps misses what seems, to this reader at least, one of the main conclusions/caveats. Many of the authors of the present manuscript have been working for a rather long time on tetraploid Arabidopsis species and have made important contributions, in particular to our understanding of the importance of genes related to meiosis during the shift from diploidy to tetraploidy. This paper is an additional contribution to this line of work. In previous studies, A. arenosa and A. lyrata were studied separately. Building on a massive dataset the authors study them jointly and highlight the importance of introgression, from other species, in the evolution of genes differentiating the two ploidy levels. Their work confirms that polyploids can act as "bridges" where

hybridization between diploids is impossible. As they point out in the discussion, this is not an isolate case and there have recently been other studies pointing out the importance of introgression to generate potentially beneficial diversity within polyploid species complexes. The aspect that may seem a bit surprising to some readers and that the authors do not stress is the fact that most genome scans studies comparing diploids and tetraploids end up finding roughly the same set of genes. Those are few and related to the same general function.

In fact, the vast majority of gene candidates from each of the genome scans comparing diploids and tetraploids are not the same. Instead, studies regularly find process-level convergence (Bohutínská et al., 2021, Molecular Biology and Evolution; Bray et al., 2024, Cell Reports). To clarify this issue, we refer to these works in our manuscript; please see lines 283-285.

Now, not so recently the prevailing views in the literature on the effect of Whole genome doubling were more in line with a "shock and awe" event than subtle and limited changes. Many recent studies have been more in line with the latter than with the former. It could be that this is simply a consequence of the methods used to compare diploids and polyploids, but in any case, I feel it would be worth pointing this out in the discussion. Are those the only changes or are we missing the rest? Although I would tend to think the latter holds, in my understanding the jury is still out.

Indeed, these are not the only changes, and we now point out to additional changes: "Recent studies have repeatedly demonstrated that certain genes involved in meiosis and cell cycle regulation are the core mediators of adaptation to WGD in A. arenosa and A. lyrata [5, 20]. Yet, additional processes, like ion management [6, 8] or pollen tube growth [15] have been found to play a role in adaptation to WGD across the family Brassicaceae, suggesting that alternative solutions to similar challenges may exist."

Minor comments:

1. line 71: removal or weakening of hybridization barriers?

Corrected to "weakening".

2. line 74. In the pre-genomic era Slotte et al. MBE 2008 was a rather convincing example of introgression from diploids to tetraploids rather than the converse.

Reference added.

3. line 159-163. Perhaps a bit more information could be lifted from the supplementary file.

We added more details about the candidate genes, but we did not move the entire text from Supplementary Text 1 to the main manuscript, as it would make this results paragraph sound like discussion. We expanded to: "We retrieved two interconnected clusters: control of cell cycle and endoreduplication, represented by the genes CYCA2;3, CYCD3;2, CYCD5;1, and chromosome pairing and segregation during meiosis, with the genes ASY1, ASY3, HEI10, PDS5b, SCC4, SDS, SYN1, ZYP1b ($p < 0.001$, Fisher's exact test; Fig. 2F, Table S3). An additional six of the 17 PSGs were not connected in this interaction network. Four of these six genes were found to be related to mRNA transcription (AT2G33845, NRPB9A, TfIIFbeta, ZOP1; Fig. 2F). AT4G18490 encodes an unknown protein that is strongly expressed in young A. thaliana flower buds and involved in cell

division [47]. AGC1.5 was not connected to any of these processes. It has recently been shown to be involved in maintaining functional pollen tubes in tetraploid A. arenosa [15]. We provide further functional interpretations of the candidate PSGs in Supplementary Text 1."

In Supplementary Text 1, we restructured and slightly expanded the text to improve its clarity: "In previous research, the role of meiosis genes in tetraploid adaptation has been identified and tested, with results suggesting that these functional changes contribute to the successful establishment of tetraploids (Yant et al. 2013; Morgan et al. 2020; Seear et al. 2020; Morgan et al. 2022). Cell cycle regulation has also been implied in relation to polyploid adaptation (Yant et al. 2013; Bray et al. 2024). Cyclins D1 and D2 have been shown to promote tolerance to genome doubling in tumors (Potapova et al. 2016; Crockford et al. 2017), while cyclin A2;3 regulates the extent of endoreduplication in A. thaliana (Imai et al. 2006), and cyclin D5;1 determines the rate of endoreduplication (Sterken et al. 2012). These findings suggest that cyclins may play a role in the adaptation to whole genome duplication (WGD), potentially by regulating endoreduplication levels, which further on influences the trade-off of cell size and cell number (Robinson et al. 2018). The role of transcription-regulating genes in the adaptation to WGD has also been proposed (Hollister et al. 2012; Marburger et al. 2019), and the transcription cycle has been shown to be linked with the cell cycle, through the interaction of RNA polymerase II with cyclin-dependent kinases (Bregman et al. 2000; Guo and Stiller 2004). It is possible that these genes interact to jointly re-time the cell cycle in response to WGD."

Reviewer #2:

This manuscript presents a comprehensive study of genetic variation contributing to repeated adaptation in autopolyploid Arabidopsis lysate and A. Arenosa relative to their diploids. The authors employed genome-wide selection scanning methods to detect the genes and processes under selection across four tetraploid lineages, and next focused on 17 PSGs to characterize types of variants. Regarding haplotypes, the authors found most tetraploid haplotypes are novel from those in diploids. Regarding SNPs, the extent of different evolutionary sources were measured: trans-specific polymorphism from diploids (65.5%), new mutations (31.9%), tetraploid-specific inter-species hybridization (71.1%). These diverse SNP sources contribute to the novel, mosaic haplotypes bearing the repeated selection signatures of tetraploid adaptation.

This is a very well written manuscript and I appreciate the concise presentation of key findings. The authors also provide thorough supplemental materials to cover technical details and additional exploration such as the functional interpretation of candidate PSGs. I would like to offer only a few suggestion for improvement:

Thank you for your encouraging comments.

* Methodological Choice: While the authors used two genome-wide selection scanning methods, it would be beneficial to discuss the rationale for choosing these methods over other popular options like McDonald-Kreitman tests. Additionally, please elaborate on the suitability of these methods for diploid-autopolyploid species.

We based our selection of methods on our extensive survey of available methods that we incorporated in a methodological book chapter that is also now referred to in the manuscript:

Bohutínská et al., 2023, Population genomic analysis of diploid-autopolyploid species, Methods in Molecular Biology. As shown in the diagram below, the level of divergence between the lineages or species compared is among the most important factors that should guide the choice of appropriate methods to identify positive selection (Oleksyk et al., 2010, Philosophical Transactions of the Royal Society B; Vitti et al., 2013, Annual Review of Genetics; Hoban et al., 2016, The American Naturalist; Booker et al., 2017, BMC Biology). As our comparison of detecting selection is within species (diploids vs. tetraploids of each of the four lineages), we selected the population differentiation-based tests instead of the substitution rate-based tests like the McDonald-Kreitman test. The McDonald-Kreitman test can be used to test for positive selection by comparing within-species nucleotide diversity and between-species nucleotide divergence. It is a powerful test when the assumption of independence between sites and fixation of causal amino acids within species is met, which is not the case in our study, as the divergence is too recent.

314 Magdalena Bohutínská et al.

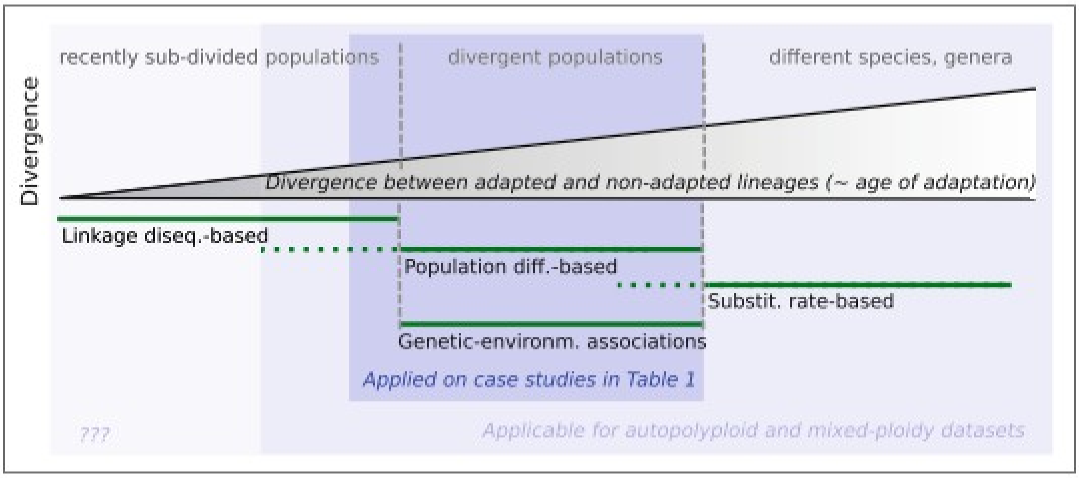

Fig. 4 Overview of selection scan methods to detect signatures of positive selection and their applicability for autopolyploid and mixed-ploidy data sets. The question marks denote so far unexplored approaches of haplotype-based selection scans

To clarify our choice of methods, we added an explanation of the principle of the methods and the reference to our standard best practice protocols for diploid-autotetraploid datasets: "To find candidate positively selected genes (PSGs) which likely contributed to adaptation to WGD in tetraploids, we employed two selection scan methods suitable for both diploids and autotetraploids following our best practices [63]. These methods are based on metrics estimated from population allele frequencies that are comparable across diploid and tetrasomic autotetraploid populations, in contrast to methods relying on assumptions on individual genotypes that are ploidy-specific [43, 63]."

* Please include data information of PacBio HiFI reads used.

Thank you for reporting this omission. We added this information as Supplementary Dataset S9. We also added the information about the assembly method to Methods.

* L183: Clarify whether the term "haplotype" refers to haplotype blocks or physical haplotypes confirmed by HiFi reads.

Thank you for spotting this inconsistency. These were haplotype blocks in most of the individuals (short-read sequenced full dataset), but each of these sequences were confirmed in its full length in our set of HiFi long read data. For the sake of simplicity, we used the summary term 'haplotype'. To avoid confusion, we now clarified this by adding "and we term them 'haplotypes' hereafter for simplicity".

* The higher proportion of trans-specific polymorphism sourced from A. arenosa compared to A. lyrata is intriguing. Besides the basal placement and larger genetic pool of A. arenosa, are there other possible explanations? For example, direction biased gene flow or selection for functional alleles from A. arenosa, etc.

We discuss different possible explanations in Supplementary Text 3: "Further, it is a pending question why there is almost an order higher contribution of candidate SNPs of A. arenosa than A. lyrata ancestry (Fig. 4A, scenario 2 and 5 vs. 3 and 6). It may partly be because diploid A. arenosa is more genetically variable then A. lyrata (pi = 0.026/0.012 for A. arenosa/A. lyrata; Table S2, (Marburger et al. 2019)). Alternatively, A. lyrata may exhibit more trade-offs between a diploid and tetraploid fitness optimum, although we miss any evidence supporting this idea. Finally, there might have been a higher initial proportion of SNPs of A. lyrata ancestry, but adaptive variation inherited from diploid A. arenosa or originated de novo might have presented a higher selective advantage. Also, some SNPs of A. lyrata origin might have become extinct in diploid A. lyrata after WGD, and thus being categorized as de novo or unsampled origin (this category of candidate SNPs is classified into scenario 4 and 7, Fig. 4A). To answer if standing variation inherited from diploid A. lyrata brought a substantial improvement in the overall adaptation of tetraploids, it would help to understand the functional impact of A. lyrata-sourced variation. Although this goes beyond the scope of this study, we found that three of the seven likely A. lyrata-sourced mutations are nonsynonymous (Asp168Gly in CYCA2;3, Ser538Pro and Met482Thr in ASY3) while four are synonymous (in PDS5b, SCC4, and ASY3), suggesting a functional impact of some, but not all of the variants."

* L448: Fig 5A should be Figure 4A

Corrected.

* L228-236: For the genomic background analysis, provide statistical test significance results from the permutation test to support the claim of lower trans-specific polymorphism, introgression, and new mutations.

Thank you for this very relevant comment. We calculated the test and added the results to the main text, which now reads: "Interestingly, however, the proportion of trans-specific polymorphism in the 232 candidate SNPs was higher compared to the genomic background average (65.5% compared to 50.0%, respectively, p-value = 0.001, 1000 permutations; Fig. 4A, Table S5). Similarly, the proportion of SNPs indicative of introgression across the tetraploid lineages was much higher for the candidate SNPs than for the average of the genomic SNP subset (71.1% compared to 56.4%, respectively, p-value = 0.002, 1000 permutations; Fig. 4A, Table S5). Finally, the proportion of SNPs exclusively present in tetraploids was slightly but nonsignificantly higher for the 232 candidate SNPs compared to the average of the genomic background (27.6% compared to 24.5%, respectively, p-value = 0.493, 1000 permutations; Fig. 4A, Table S5)."

For the reviewer's interest, here we also show the histograms underlying the permutation test:

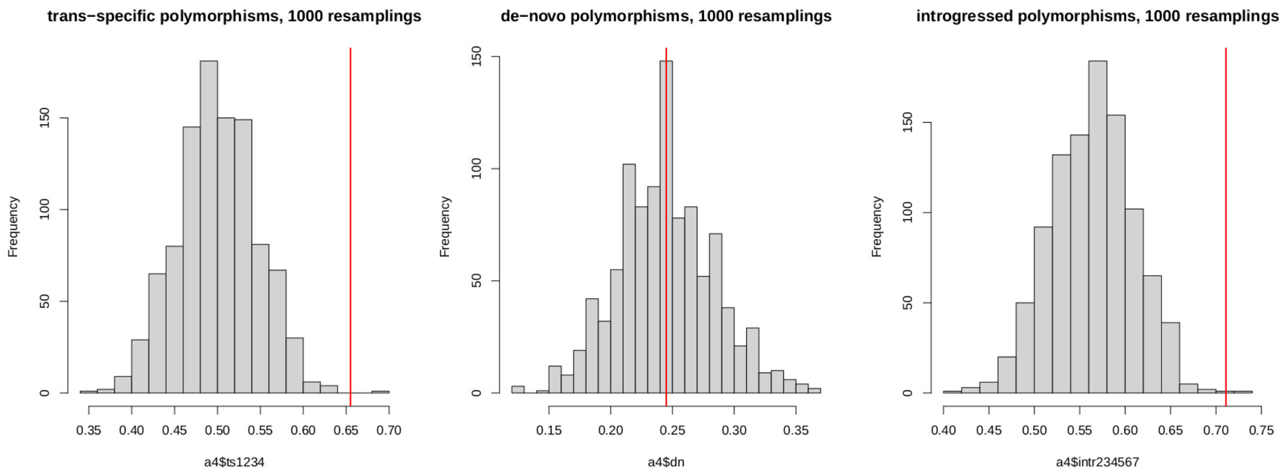

* Only 11 PSG sequences were provided in TableS5.

Thank you for pointing out this omission. We corrected the Supplementary materials and now report sequences for all 12 PSGs as Supplementary Dataset S5.

Reviewer #3:

This is a compact, well-written manuscript presenting some very interesting results. For me it was a good wake-up call to be more attentive to the Arabidopsis polyploidy system than I have been. Despite knowing some of the story from these authors concerning evolution involving meiosis-related genes in polyploids, it is clear that I am woefully behind in reading about the overall Arabidopsis evolutionary picture, particularly the issue of trans-specific variation involving wide hybridization. That, of course, plays a key role in the research reported here. The population genomic resources used in the study are quite impressive—the envy of those not working on near-model species—and are leveraged to excellent effect in this study.

Thank you for your supportive comments on our work.

The authors report that autopolyploids classified as A. lyrata or A. arenosa, though bearing different names, share haplotypes through post-polyploidy hybridization/introgression. That is a very interesting finding, one that I was more familiar with from other systems and from theoretical considerations, nicely demonstrated here.

Next, they identify 17 genes that are under positive selection in the polyploids (PSGs); these are either related to meiosis or to other cellular-level phenomena (notably cell cycle). This makes sense given previous work. What I found (find) difficult to understand fully was the emphasis, particularly in the Supplementary text, on endopolyploidy. I do not know the literature on the significance of endopolyploidy in natural Arabidopsis populations that is cited here. I do know something about endopolyploidy in synthetic A. thaliana polyploids (e.g., Corneille et al. 2017, Plant Physiology; Robinson et al. 2018, Plant Cell), but here the argument seems to be that the degree of endopolyploidy is associated with adaptation. This should be explained better (see comment below), but I seem to have gotten the main interesting points of the study without understanding this fully—there are 17 genes that show signs of positive selection in the polyploids

in a way that is consistent with adaptation to an altered cellular landscape in which function is challenged by the presence of a doubled genome.

Thank you for pointing out this discrepancy. In the context of polyploidy adaptation in A. arenosa/lyrata, the emphasis has largely been put on meiosis and related processes in the past, and only recently it has been shown that other important processes such as pollen tube growth (Westermann et al., 2024, Science) are also likely under compensatory evolution after WGD. In light of these findings, we aimed to highlight an additional process that has so far been largely neglected by the Arabidopsis polyploidy literature, endoreduplication, that shows a contrasting phenotype across ploidies and may be linked with some of the presented PSGs.

To better clarify this, we (i) explained possible selective forces promoting the reduction of the degree of endoreduplication in established tetraploids and (ii) highlighted that the link between the observed relevant PSGs and the endoreduplication phenotype is correlative, and further investigations are needed to prove causality and possible fitness advantages of changes in the endoreduplication phenotype in polyploids. The text now reads:

"Here, we investigated whether endoreduplication follows a similar pattern of compensatory evolution after WGD, in line with the suggestion that "whole-plant polyploidy may have a subtle inhibitory effect on the extent to which cells undergo endopolyploidy" [1]. Using flow cytometry, we found a substantial decrease in endoreduplication within established tetraploids compared to synthetic tetraploids, while accounting for the variation in diploids (Supplementary Text 2, Fig. S4, Fig. S5). This finding suggests compensatory changes, possibly reflecting a high cost of additional rounds of genome duplication in polyploids and/or structural constraints on maximum cell size [48, 49]. It should be highlighted, however, that causality between the observed phenotype and variation in the relevant PSGs needs further functional validation."

The authors then show that for the 12 genes with sufficient SNPs, tetraploids possess a major haplotype not found in diploids, including putative diploid source populations. Strikingly, these tetraploid-specific haplotypes have sequences that are a mosaic of different sources—not only the two diploid cytotypes of the same two species whose names the polyploids bear or from de novo mutations in the tetraploids, but in a significant percentage of cases coming from other Arabidopsis species. SNPs in each gene are traced to their sources in one of 7 scenarios. Fig. 4C is very helpful in illustrating what was done to produce the data summarized in 4B; 4A took a bit of time to decipher, but I don't have any good suggestion of how to make it more clear (I think part of the problem may be my interpretation of "standing in diploids", which can mean as few as a single species, and might be clearer with a longer description such as "standing in one or more diploid species" … but there isn't much room in this already crowded, compact figure). Networks for each gene are presented in Supplementary Fig. 7, which I found very useful (see comment below).

We described "standing in diploids" in the figure caption, but do not say "diploid species", as this may be confusing in the case of A. arenosa and A. lyrata, where both species are not only diploid.

One thing that seemed lacking here was an indication of where the SNPs under selection, identified in the "candidate SNP" approach that identified all of the PICMIN genes plus three additional candidates. Presumably at least some of these are among those shown for the two genes in Fig. 4C,

and all of them can be placed in one of the 7 scenarios. I thought I might find that information in Supplemental Dataset 3, but (as noted below) I found that spreadsheet undecipherable.

We agree that this was difficult to follow and not well indicated in Supplementary Dataset S3 (which is now Dataset S4 due to a different order of the supplementary datasets). We added a title to all Supplementary Datasets and further explained the content of Dataset S4 in its caption*: "Candidate SNPs were used in the selection scan to identify positively selected genes (PSGs), following a three-step procedure. First, 1% outlier SNPs were identified; second, the top quartile of genes with the highest density of outlier SNPs were identified; and third, these genes were overlapped among the four tetraploid lineages of A. arenosa and A. lyrata* to identify repeatedly differentiated genes*. The PSGs and their overlap are shown here."*

In the Discussion, the authors present a narrative involving origins of the two polyploids and subsequent introgression, with selection on genes contributing to meiotic (or more broadly, cytological) stability. I have already commented on my inability to understand how this relates to endopolyploidy, which is a perfectly natural part of the normal functioning of some plant cell types. It seems to me that the endopolyploidy part is—or at least comes across as—a rather peripheral aspect of this paper, disconnected from the major points that are made.

We agree with this and made modifications accordingly. Please see our response to the aspect of endoreduplication in our response to your first point.

In lines 311-312 the authors state, "Altogether, we demonstrated that extensive reshuffling of trans-specific, species-specific, and novel variation occurred in response to the challenges of WGD." Most of this seems well-supported by the results, but "in response to" sounds rather teleological, as though the polyploid was looking for a way to adapt. Perhaps I am being too critical, but I wondered at one point whether genes other than the 12 PSGs studied here are also mosaics. If so, then the combining of various sources of variation is not "in response to" anything in particular—it simply is a product of the biology of these *Arabidopsis* lineages. The authors may be admitting the same thing by saying that this kind of mosaicism is not confined to polyploids, but also in diploids (lines 315-316) where clearly it is not "in response to the challenges of WGD", at least not in the immediate past. Given the variation in the distribution of sources of SNPs across the 12 genes (Fig. 4B), and the similarity of the "genome" distribution in the same figure, I would be surprised if non-PSGs didn't show the same range of variation in terms of sources of SNPs.

We corrected this misleading sentence to reflect that we do not imply mosaicism to be a direct consequence of adaptation to WGD. The sentence now reads: "Altogether, we show that extensive reshuffling of trans-specific, species-specific, and novel variation occurred after WGD."

None of this diminishes the value of this work, which I think is of considerable interest, and as best as I can tell has been executed competently. I think it will be a useful contribution to the area of polyploid evolution.

Thank you very much!

Specific comments, by line number:

Line 59. “Autopolyploid” is here defined in the commonly used taxonomic sense, which I would argue is genetically misleading. There is an excellent very recent paper on this topic from Anneliese Mason (one of the stars in the polyploidy field) that I think the authors may want to cite here:

Lv, Z., Addo Nyarko, C., Ramtekey, V., Behn, H., and Mason, A.S. (2024). Defining autopolyploidy: Cytology, genetics, and taxonomy. American Journal of Botany 111, e16292.

Thanks for pointing this out; we have added it, as well as a review by Kirsten Bomblies. It now reads: “Over the last decade, a body of work has accrued describing means by which within-species WGD lineages (taxonomic autopolyploids) or WGD lineages with polysomic inheritance (random chromosome pairing; genetic autopolyploids; [3, 4]) overcome these challenges.”

117ff. I’m a bit puzzled by the lack of any evidence of reticulation in the TreeMix figures (2B, 2C); this isn’t a program I have used recently, but my recollection is that it is used to show gene flow (e.g., via introgression), which is clearly shown later in Fig. 2D. The legend has no explanation of how to interpret the topologies of these figures, or perhaps the lack of cycling in the graph.

*We clarified this point that may indeed have been confusing. In our set of TreeMix analyses we initially allowed 0 to 5 migration events. However, as our primary aim for this analysis was presenting the topology of each species separately, but with bootstrap support, we preferred presenting the basic 0 migration tree over the tree with migration edges *within* each species. The reason is simply that the trees with additional migration edges do not allow bootstrapping (Pickrell & Pritchard, 2012, Plos Genetics).*

*In contrast to the above, Fig. 2D shows a complementary finding to Fig. 2B and Fig. 2C, namely gene flow *between* the tetraploids of both species. This finding is supported by the TreeMix analysis below (not shown in the article). To clarify this, we added to the figure caption: “Phylogenetic relationships among the focal populations of A. arenosa (B) and A. lyrata (C) inferred by TreeMix analysis assuming no migration.”*

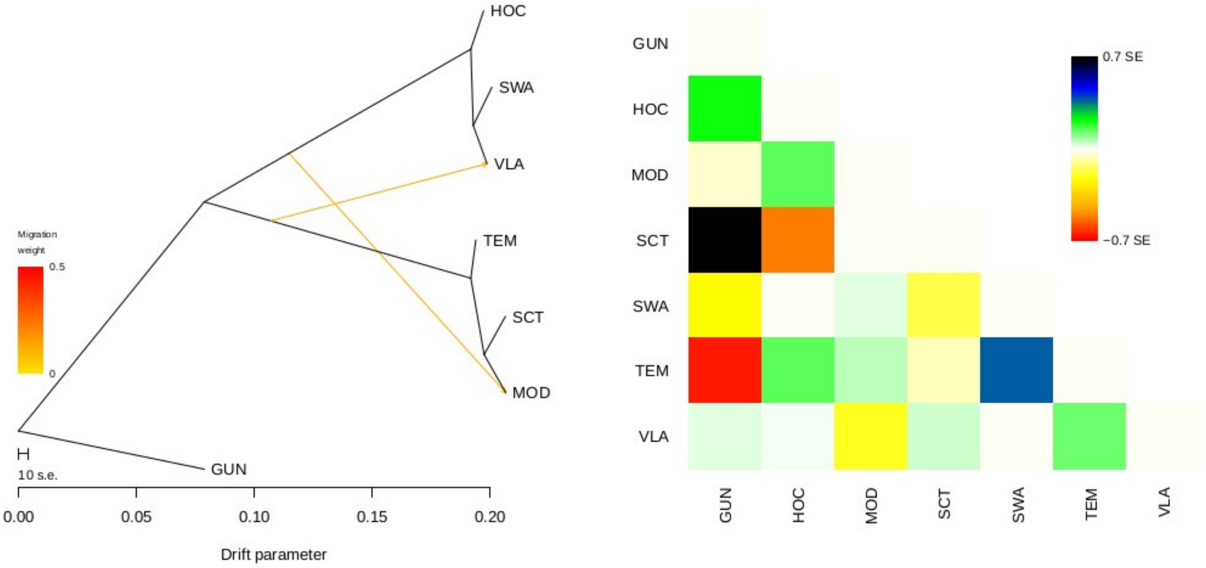

148. Dataset 3. I looked at this spreadsheet, and could not make much sense of it. It would be very helpful if an explanation of some kind was included somewhere, preferable at the top of the file as in a regular text table.

We added this explanation now; please see our explanation above.

159-160. “PSGs associated with the cell cycle trait endoreduplication on tetraploids”: This is awkwardly phrased. Maybe add commas or quotes?

We rephrased as follows: “Here, we investigated whether endoreduplication follows a similar pattern of compensatory evolution after WGD, in line with the suggestion that “whole-plant polyploidy may have a subtle inhibitory effect on the extent to which cells undergo endopolyploidy” [1]. Using flow cytometry, we found a substantial decrease in endoreduplication within established tetraploids compared to synthetic tetraploids”

173-174. “tetraploids aggregate as mosaics from different allelic sources (Fig. 1B)”:

Done.

250. “polyploidy-mediated hybrid seed rescue” is not a term with which I am familiar, at least by name. I took a quick look at Ref. 15 to see what is meant, and found that it relates to endosperm balance number. If there is a way to explain the phenomenon succinctly here instead of using what is a rather jargon-ish term, that could be helpful to readers like me.

We changed to: “diploids of both species exhibit strong postzygotic barriers, whereas polyploidy-mediated hybrid seed rescue (i.e. reestablishment of endosperm cellularisation [17]) enables hybridization between tetraploid A. arenosa and A. lyrata [20, 38].”

267-268. “We found a substantial decrease in endoreduplication within established tetraploids compared to newly synthesized tetraploids.”: What is the adaptive significance of reduced endoreduplication? It is a normal part of development in many species, in which case it presumably is beneficial. My sense from the literature is that the effect of whole-plant doubling on endopolyploidy is variable.

We embedded our finding of reduced endoreduplication within the context of compensatory evolution following WGD, without speculating much about its adaptive significance: “Here, we show signals of such compensatory evolution also in the case of endoreduplication, as established A. arenosa tetraploids exhibited a decrease in endoreduplication compared to newly synthesized tetraploids. Additional mechanistic work is required to understand the functional and strategic basis of this shift. It may be a homeostatic response to maintain cell size, overall DNA content, or mitotic stability at high ploidies [48, 49].”

299. “retainment”: Probably "retention" would be a better word here.`

Corrected.

343-344. “Arabidopsis arenosa and A. lyrata autotetraploids show random segregation of chromosomes [32, 38], resulting in allele frequency estimation comparable to diploids”: Random

segregation in an autopolyploid should produce tetrasomic ratios, not disomic ratios as in diploids. Consequently I'm not sure what is meant here. See Lv et al. (2024) for discussion of the relationships among segregation ratios, bivalents/multivalents, and auto/allopolyploidy.

Corrected, we explain: "Arabidopsis arenosa and A. lyrata autotetraploids show random segregation of chromosomes with a tendency towards bivalent formation [5, 20, 35, 39]. Bivalent formation during metaphase I of meiosis in established autotetrapolyploids results from reduced crossover frequencies relative to neo-tetraploids or diploid relatives [3]. This results in allele frequency estimation comparable to diploids [67]."

Supplementary text 1: "In summary, these results point to an adaptive compensation for DNA content per nucleus: while nearly double in neo-tetraploids, it returns toward diploid levels in established tetraploids (Fig. S4)." Genome downsizing references (e.g., Leitch and others) could be useful here; this is not a new concept in the polyploidy literature.

We added: "while nearly double in neo-tetraploids, it returns toward diploid levels in established tetraploids (Fig. S4), in line with genome downsizing after a WGD event (Leitch and Bennett, 2004).
